# Thyroid hormone dysfunction during pregnancy: A review

**Published:** 2016-11

**Authors:** Aynadis Alemu, Betelihem Terefe, Molla Abebe, Belete Biadgo

**Affiliations:** 1 *Amhara Regional Health Bureau, Dessie Regional Health Research Laboratory, Dessie, Ethiopia*; 2 *School of Biomedical and Laboratory Sciences, College of Medicine and Health Sciences, University of Gondar, Ethiopia.*

**Keywords:** *Thyroid hormone*, *Pregnancy*, *Gondar*, *Ethiopia*

## Abstract

Thyroid dysfunctions such as hypothyroidism, thyrotoxicosis and thyroid nodules may develop during pregnancy leading to abortion, placental abruptions, preeclampsia, preterm delivery and reduced intellectual function in the offspring. Epidemiological data have shown the significant role of maternal thyroid hormone in fetal neurologic development and maternal health. It has been suggested that the deleterious effects of thyroid dysfunction can also extend beyond pregnancy and delivery to affect neuro-intellectual development in the early life of the child. Pregnancy poses an important challenge to the maternal thyroid gland as hormone requirements are increased during gestation as a result of an increase in thyroid- binding globulin, the stimulatory effect of HCG on TSH receptors, and increased peripheral thyroid hormone requirements. Maternal thyroid dysfunction is associated with increased risk for early abortion, preterm delivery, neonatal morbidity and other obstetrical complications. Early diagnosis for thyroid dysfunction of pregnant women and treatment of thyroid dysfunction during pregnancy is important and cost effective to avoid both fetal and maternal complications secondary to thyroid dysfunction. Therefore the aim of this review was to assess the thyroid function changes occurring during pregnancy, the different disorders with their maternal and fetal implications, the laboratory diagnosis and the best ways of management of these conditions.

## Introduction

Pregnancy is a natural physiological changes that is accompanied with hormonic and metabolic alterations caused by a variety of conditions that results in many pathophysiologic processes, some of which have potentially serious outcomes if left untreated. Thyroid diseases during pregnancy are related to maternal and fetal complications. Therefore in this article, we try to review the thyroid function aberrations occurring during pregnancy, the different disorders with their maternal and fetal implications, the laboratory diagnosis and the best ways of management of these conditions.

## Physiological changes of thyroid function in mother and fetus during pregnancy

Thyroid hormones (TH) are very important for growth and development of brain for the fetus and neonate, in addition for many other aspects of pregnancy, fetal growth and development (1). The thyroid gland dysfunctions like hypothyroidism and thyrotoxicosis can affect the mother health as well as the child before and after delivery that can result in fetal disease; in humans, this includes a high incidence of mental retardation (2). The fetal thyroid gland begins concentrating iodine and synthesizing THs after months of gestation. Although the requirement for TH before this time is exclusively supplied by the mother which is most important to fetal brain development, significant fetal brain development continues considerably beyond the first trimester (1, 2). Evident maternal thyroid failure during the first half of pregnancy has been associated with several pregnancy complications including preeclampsia, premature labor, fetal death and low birth weight and intellectual impairment in the offspring (3). 

THs have most profound effects on the terminal stages of fetal brain differentiation and development, including synaptogenesis, dendrites growth and axons myelination and neuronal migration. TH receptors are broadly dispersed in the fetal brain, and existing prior to the time the fetus is able to synthesize TH. Evidence has confirmed that it is challenging to identify the molecular targets for TH action in the developing brain, but some improvement has been made (4, 5). 

## Mechanisms of physiological changes of thyroid function in pregnancy

Thyroid stimulation starts as early as the first trimester by β-HCG hormone, which shares some structural homology with thyroid-stimulating hormone (TSH). There is also an estrogen-mediated increase in circulating levels of thyroid-binding globulin (TBG) during pregnancy by 2-3 times in serum TBG concentrations. TBG which is one of the numerous protein that transport TH in the blood with high affinity for thyroxine (T4) increases in serum a few weeks after conception and ranges a plateau during mid-gestational period. The mechanism for this increase in TBG involves both increased hepatic synthesis of TBG and estrogen mediated perpetuation in sialylation of TBG that increases the half-life from 15 min to 3 days to fully sialylated TBG (6).

Elevated levels of TBG lead to lowered free T4 concentrations, which results in increased TSH secretion by the pituitary and, subsequently, enhanced production and TH secretion. The net effect of elevated TBG synthesis is to force a new equilibrium between free and bound THs and therefore a substantial increase in total T4 and triiodothyronine (T3) levels. The augmented demand for THs is reached by about 20 wk of gestation and persists until term (6, 7).

Reflecting changes in iodine metabolism, which is an essential requirement for TH synthesis, increased demand for iodine results from a significant pregnancy-associated increase in iodide clearance by the kidney and draw off maternal iodide by the fetus. During pregnancy there is an increased iodine excretion in the urine as a result of increased glomerular filtration and decreased renal tubular absorption. In addition, maternal iodine is actively transported to the feto-placental unit, which contributes to a state of relative iodine deficiency (8, 9).

The other factor is the impact of HCG secreted by the placenta of humans. Thyroid stimulation in response to the thyrotropic activity of HCG overrides the normal action of the hypothalamic pituitary thyroid feedback system. TSH that can bind and transduce signaling from the TSH receptor on thyroid epithelial cells. Closure to the end of the first trimester of pregnancy in humans, when HCG levels reaches at peak, a substantial fraction of the thyroid-stimulating activity is from HCG. During this time, blood levels of TSH become suppressed. The thyroid-stimulating activity of HCG actually causes some women to cause transient hyperthyroidism (8, 9).

The potential source of TH for the fetus is its own thyroid and the thyroid of the mother. Human fetuses acquire the ability to synthesize TH at approximately the first trimester of gestation. Current evidence from several species indicates that there is significant transfer of maternal THs across the placenta and also the placenta contains deiodinases that can convert T4 to T3 (10). Pregnant mother and infant protection is a priority in the health because these population groups are mostly exposed to the diseases and death. Thyroid dysfunction is one of the common complications of pregnancy and it contributes significantly to the maternal and fetus morbidity and mortality. There is limited attention and information related to thyroid dysfunction and its complication during pregnancy. Therefore, the aim of this review was to assess the magnitude of maternal and fetal thyroid dysfunction and its complication in pregnancy secondary to thyroid dysfunction.

## Thyroid hormone abnormalities during pregnancy

Hyperthyroidism

Hyperthyroidism typically is the disease process in which excessive TH is synthesized and excreted, whereas the term thyrotoxicosis refers to increased amounts of TH in the circulation (11). It can occur in approximately 1% of the population and up to 0.4% of the pregnancies (12). Previous study by Wang *et al* found that the prevalence of thyroid dysfunction was 10.2%, hyperthyroidism, hypothyroidism and hypothyroxinemia were 1.8, 7.5 and 0.9% respectively (13).

There are 2 causes of hyperthyroidism, like classic causes which is found in the general population, and the other causes are specific during pregnancy. True hyperthyroidism is differentiated from other forms by elevated radioactive iodine uptake (RAIU). The other forms are differentiated from true hyperthyroidism by decreased RAIU like hyperthyroidism caused by factors other than thyroid gland over-activity may result from inflammatory thyroid disease, pregnancy-specific associations (like hyperemesis gravidarum and hydatid form mole) and the presence of ectopic thyroid tissue or by exogenous sources of TH (14-17). 

Tissue effects of hyperthyroidism include accelerated metabolism, suppressed serum TSH, low serum cholesterol, increased bone turnover and reduced bone density with an increased risk of osteoporosis and fracture (18). A study by Marvisi *et al* found that hyperthyroidism strongly is associated with lower TSH values and increased pulmonary arterial pressure which leads to severe pulmonary hypertension (19). In the first trimester of gestation, the normal elevation in total T4 and total T3, due to estrogen-induced increase in TBG concentration and HCG thyroid stimulation with suppression of serum TSH, may pause difficulties in the diagnosis of maternal hyperthyroidism (20).

## Characteristics of various types of hyperthyroidism

**TSH-induced hyperthyroidism** **:** This type of hyperthyroidism is due to dysfunction in TSH that regulates T3 and T4 production. Increased stimulation by inappropriate TSH secretion may be caused by TSH-secreting pituitary adenomas that influences elevated TH synthesis and release, and not responsive to normal hormonal feedback control. Diagnosis is confirmed by demonstrating a lack of TSH response to thyrotropin releasing hormone (TRH) stimulation and radiologic imaging of the pituitary (21). 

**Pituitary resistance to thyroid hormone: **It refers to resistance of the pituitary to TH feedback control, possibly resulting from receptor modification. Diagnostically these patients display an appropriate increase in TSH in response to TRH and suppressed TSH in response to T3 (14). 

**Hyperthyroidism from thyroid stimulators other than TSH** **: **Graves’ disease (GD) is the most common cause of hyperthyroidism which is an autoimmune syndrome resulting from the production of thyroid stimulating antibodies (TSAbs), sometimes called TSHR Ab capable of stimulating thyroidal TSHRs, resulting in excessive TH production and release, and overstimulation of gland growth. Autoantibodies that react with the orbital muscle of the eye and fibroblasts of skin are also produced and initiate the extra thyroidal manifestations of GD (22, 23). 

Like other autoimmune diseases, the activity of GD is aggravated during the first trimester of gestation and decreased during the latter half of pregnancy, to be aggravated again in the first few months after delivery or late in the postpartum period (24, 25). Fetal effects of Graves hyperthyroidism are hyperthyroidism, intrauterine growth, retardation, short gestational age, still birth, craniosynostosis and also maternal effects are hypertension, preeclampsia, preterm delivery, heart failure, thyroid crisis and placental abruption (26). 

**Trophoblastic disease: **It is resulted from the production of thyroid stimulators other than TSH. It is a general term that includes benign and malignant conditions of hydatidiform mole with frequency of approximately 1 in 1500-2000 pregnancies and choriocarcinomas with frequency of 1 in 40-60000 pregnancies (27, 28). The etiology of the hyperthyroidism is thought to be related to the increased levels of serum HCG in these patients (15). HCG is secreted by placenta from early pregnancy that shares a common alpha subunit with TSH. HCG stimulates the normal maternal thyroid via TSH receptor to synthesize and secrete TH (29). 

Hyperthyroidism attributable to trophoblastic disease should be suspected in patients who demonstrate increased free thyroxine (FT4) and free triiodothyronine (FT3) levels, decreased TSH, and significantly increased HCG (30). Although FT4 and FT3 levels can be increased with HCG levels >50,000 IU/L in patients with trophoblastic tumors, serum HCG may reach >300,000 IU/L compared to the >50,000 IU/L seen in normal pregnancy (30). Thyrotoxic patients also have a higher serum T4 to T3 ratio than Graves’s patients (28). 

**Gestational thyrotoxicosis: **Gestational thyrotoxicosis is a transient increase in thyroid secretion leading to thyrotoxicosis of varying degrees of severity that can occur when HCG levels are very high and considered as a fairly normal phenomena. As HCG is a thyroid stimulator, a state of hyper stimulation of the thyroid gland is common in early pregnancy. This form of hyperthyroidism differs from GD in that occurs in women without a past history of GD and in the absence of detectable TSHR-Ab (29, 31). This disorder occurs in 0.2% of pregnancies. Mostly patients with hyperemesis gravidarum, as many as 60% exhibit hyperthyroidism (32, 33). Interestingly, more severe vomiting is associated with a greater degree of thyroid stimulation and higher level of HCG. On laboratory examination, the serum FT4 is more frequently increased compared with the serum FT3 level (34). 

The clinical importance of gestational transient thyrotoxicosis (GTT) has probably been overlooked. From recent studies, it is now thought that the prevalence of GTT may be as high as 2-3% of all pregnancies that is 10-fold more frequent than hyperthyroidism due to GD (35). The syndrome GTT is important to differentiate from GD because the course of both conditions, the associated fetal risks, and the management and follow-up are entirely different (36, 37). 

**Hyperthyroidism from thyroid** **gland autonomy: **Toxic adenoma, as an autonomous thyroid nodule, is a discrete thyroid mass whose function is independent or normal pituitary control. These nodules may be toxic adenomas or hot nodules based on their uptake on radioiodine and appearance on a radioiodine thyroid scan. Toxic or hot nodules secrete THs independent of the pituitary because this tissue contains mutated TSHRs. Thyroxine levels typically are elevated in these patients sometimes only T3 levels are increased (38). Consequently if T4 concentrations are normal in such patients T3 levels should be determined to rule out T3 toxicosis. 

In toxic multinodular goiter, the thyroid gland normally enlarges in reaction to an increased demand for THs that occurs in pregnancy, iodine deficiency and immunologic, genetic disorders. During these phenomenon, there is increased TSH secretion and a compensatory increase in thyroid follicles and TH synthesis. When the situation for excessive production of TH subsides, TSH secretion decreases and the thyroid gland returns to normal size. However, permanent changes may have arisen in some follicle cells that can function autonomously relative to TSH. These autonomous follicles may produce excessive TH unregulated by TSH, resulting in thyrotoxicosis symptoms similar to GD without infiltrative ocular manifestations or myxedema (14, 38). 

**Thyrotoxicosis associated with inflammatory thyroid disease** **: **Subacute thyroiditis also called de Quervain’s thyroiditis, is a painful condition of the thyroid gland that appears to be due to viral invasion of the thyroid parenchyma and begins much more suddenly than Hashimoto's thyroiditis. Postpartum thyroiditis may be subclinical or produce only fragile clinical manifestations. If it is still present, thyroid function testing will show elevated TH levels and a suppressed TSH level. After the thyrotoxicosis decreases, the patient will be euthyroid for a few weeks. Then, because the thyroid has tired its store of hormone, hypothyroidism will develop. In response, the TSH level begins to rise in about one month, normal thyroid function is usually restored, rarely, the hypothyroid phase may be prolonged by 3-5 months, but rarely it is permanent (39, 40). 

Thyrotoxicosis caused by postpartum thyroiditis may be clinically identical to GD, which subsides during pregnancy and worsens immediately afterwards. The distinguishing feature is that in patients with GD, the thyroid actively produces hormone and so takes up radioiodine at 3-5 times the normal rate. On the contrary, the thyroid gland releases hormone into the serum in patients with postpartum thyroiditis (40).

## Ectopic thyroid tissue

**Struma ovarii and follicle cancer** **: **A teratoid tumor of the ovary that is capable of producing TH is strauma ovarii. This is an extremely rare form of thyrotoxicosis and is evident by hyperthyroidism without thyroid gland enlargement and suppressed RAIU. The disease can be detected by whole body scanning with RAI. Both surgery and radioiodine therapy is required since the tissue is potentially malignant. TH with sufficient preserved function can be secreted to cause thyrotoxicosis in case of metastatic follicular carcinoma. In most of these cases there was a previous diagnosis of thyroid malignancy (21).

**Risks of hyperthyroidism on fetal and maternal well-being** **: **Elevated levels of TH complicating pregnancy is not common, but potentially severe condition occurs in about 2 out of 1000 pregnancies (41). Uncontrolled hyperthyroidism during pregnancy can promote to congestive heart failure, preeclampsia, rise in blood pressure in late pregnancy, thyroid storma, miscarriage, premature birth and low birth weight (42). Hyperthyroidism in a newborn can result in rapid heart rate, which may lead to heart failure; early closure of the soft spot in the skull; poor weight gain; irritability; and sometimes an enlarged thyroid that can press against the windpipe and interfere with breathing (29). Autonomous production of TH and inadequately treated maternal hyperthyroidism may result in fetal and neonatal hyperthyroidism due to the trans-placental transfer of stimulatory TSHRAb (43, 44).

Clinical neonatal hyperthyroidism occurs in about 1% of infants born to mothers with GD. Not often neonatal hypothyroidism may also be observed in the infants of mothers with Graves’ disease, this may result from trans placental transfer of circulating maternal anti-thyroid drugs, pituitary-thyroid axis suppression from transfer of maternal T4 (45). Thyroid storm the most severe manifestation of hyperthyroidism results from untreated hyperthyroidism and may be precipitated by infection trauma, a surgical procedure and diabetic ketoacidosis (42, 46). Thyrotoxic periodic paralysis is another uncommon problem of hyperthyroidism. It is a reversible disorder characterized by acute muscle weakness and hypokalemia. The attacks of periodic paralysis are precipitated by hypokalemia that is caused by a transcellular shift rather than total body depletion of potassium (46). 

**Diagnosis of hyperthyroidism** **: **Measurement of the TSH level is the only initial test necessary in a patient with a possible diagnosis of hyperthyroidism without evidence of pituitary disease. If the TSH level is low, then FT4 should be measured to evaluate for thyrotoxicosis. Measurement of FT3 is helpful in the clinical diagnosis of thyrotoxicosis when the FT4 values are unexpectedly normal (47). Thyroid-stimulating antibody levels can be used to monitor the effects of treatment with anti-thyroid drugs in patients with GD (48). High iodine uptake is seen in disease that cause increased T4 synthesis, including GD, toxic multinodular goiter, toxic adenoma and molar pregnancy. Low iodine uptake is seen disease that causes inflammation and release of T4 including subacute thyroiditis, thyrotoxicosis factitia, iodine ingestion and post-partum thyroiditis (21, 49).

**Treatment of hyperthyroidism during pregnancy** **: **Maternal and fetal outcome is directly associated to control of hyperthyroidism. During pregnancy, mild hyperthyroidism, in which TSH is low but FT4 is normal, does not need treatment. More severe elevated TH concentration is treated with anti-thyroid medications. The medications cross the placenta in small amounts and can decrease fetal TH production, so the lowest possible dose should be used to avoid hypothyroidism in the baby. The medications can cause adverse effects in some people, like allergic reactions, patients with leucopenia, which can lower a person’s resistance to infection, liver failure, in rare cases (50, 51). Molar disease should also be considered and can potentially lead to fulminant hyperthyroidism, particularly in women with a pre-existing autonomous nodular goiter. However, uncomplicated hydatidiform mole is now easily diagnosed in the early stages of gestation and therefore, will rarely lead to severe hyperthyroidism, because it lasts only for a few weeks or months and is rapidly cured by the removal of the pathological trophoblast (51).

## Hypothyroidism

It is the most common pathological deficiency of TH that accounts approximately 2% of women and 0.1-0.2% of men (52). It is common in pregnancy with a predictable prevalence of 2-3% and 0.3-0.5% for subclinical and overt hypothyroidism respectively (53). Endemic iodine (I^-^) deficiency accounts for most hypothyroidism in pregnant women worldwide while Hashimoto’s disease is the supreme cause of hypothyroidism in I^-^ adequate parts of the world (54).

Manifestations of hypothyroidism can range from asymptomatic subclinical detection to overt myxedema, which is rarely seen due to widespread screening for thyroid disease (55). A cross sectional study conducted by Alkafajei *et al* found that the prevalence of sub clinical hypothyroidism was 4.3% and 20.8% according to the general laboratory and internationally adopted criteria respectively (56). Effects of hypothyroidism on the fetus like impaired brain development, intrauterine death, low birth weight, neonatal respiratory distress, increased fetal distress and preterm birth are commonly observed (57, 58). A study also reported by Renée *et a*l and Negro *et al* showed that infants born to thyroid peroxidase (TPO) antibody positive mother and euthyroid pregnant women had significantly smaller head circumference, reduced brain weight and lower brain-to- body ratio than those born to TPO antibody negative mothers which is associated with an increased risk of miscarriage and premature delivers (57, 59). 

## Characteristics of various types of hypothyroidism

**Hashimoto’s disease (Chronic autoimmune thyroiditis)** **:** It is the most common type of thyroiditis and the most common cause of primary hypothyroidism. In which the immune system attacks the thyroid, causing inflammation and interfering with its capacity to produce THs (25). Hashimoto's thyroiditis patients may develop a goiter or have thyroid atrophy. Patients with goiter may have antibodies that stimulate thyroid growth, whereas patients with an atrophic thyroid have antibodies that inhibit the trophic effects of TSH on the gland. Early Hashimoto’s thyroiditis is associated with a firm goiter, but later in the disease process a shrunken fibrotic hypo functioning thyroid gland develops (60, 61). Chronic autoimmune thyroiditis is the most important cause of hypothyroidism in up to 90% of pregnant women with hypothyroidism during pregnancy test positive for thyroid antibodies (53). 

The disease is characterized by the presence of high affinity and concentrations of serum thyroid antibody. The most frequently detected antibodies are TPO antibody and thyroglobulin antibody (TGAb). In its initial phase, Hashimoto’s thyroiditis can cause hyperthyroidism that presents as a painless goiter caused by lymphocytic infiltration of the thyroid gland. Positive TPO antibody and/or TGAb test results are found in approximately 5% of euthyroid pregnant women. However, a thyroid autoantibody prevalence of up to 15% has been found in pregnant women (62).

**Postpartum thyroiditis** **: **It is an inflammation of the thyroid gland that affects about 5-18% of healthy pregnant women in different populations during the first year after delivery (63). The hypothyroidism of these disease states results from inflammation secondary to infiltration of the gland by lymphocyte and leucocytes. Women have transient hyperthyroidism or hypothyroidism occurring 3-6 months after delivery that is associated with the development of a small, painless goiter. More than 90% of patients with thyroiditis recover wholly but the condition may relapse during second pregnancy. It should be noted that patients with postpartum hypothyroidism may present with postpartum depression. As much as 30% of cases after 3 years, and in 50% at 7-10 years develop permanent hypothyroidism. Nearly all the women with postpartum thyroiditis have TPO antibody. This marker can be a useful screening test in early pregnancy as 50% of women with antibodies will develop thyroid dysfunction postpartum (64). 

**Iatrogenic hypothyroidism** **: **Iatrogenic hypothyroidism is resulted from thyroid surgery, exposure of the thyroid to external radiation for neck carcinomas or from RAI to treat GD. Typically hypothyroidism occurs within 1 month following total thyroidectomy and within 1 year after RAI therapy for GD (14). 

**Iodine deficiency, thyroid enzyme defects, thyroid hypoplasia and goitrogens** **: **Iodine deficiency or excess, and the ingestion of goitrogens may cause hypothyroidism on rare occasions by decreasing TH synthesis or release. Iodine deficiency, thyroid enzyme defects, thyroid hypoplasia and goitrogens may cause TH deficiency in a developing fetus, resulting in cretinism (14, 65). A study by Mezosi *et al* reported that the mean thyroid volume of women with severe iodine deficiency was significantly larger than that in the group with adequate iodine intake and the frequency of goiter was increased in all groups with iodine deficiency (66).

**Congenital hypothyroidism (CH)** **: **Congenital hypothyroidism (cretinism) is a TH deficiency at birth that occurs in 1/3000 newborns, as a result of the absence of thyroid tissue (thyroid dysgenesis) and hereditary defects in TH biosynthesis. Thyroid dysgenesis occurs more commonly in female infants and permanent abnormalities occur in 1 of every 4000 infants. It is one of the most common preventable causes of mental retardation. Most infants with CH do not show an obvious clinical manifestation of hypothyroidism at birth. This may result from remaining neonatal thyroid function, because of an over expression of deiodinases by compensatory mechanisms in target organs, or in the TH received from breast milk (67). 

**Risk of hypothyroidism on fetal and maternal well-being** **: **Some similar problems caused by hyperthyroidism can occur with hypothyroidism. Uncontrolled hypothyroidism during pregnancy can lead to preeclampsia, anemia, abruption, miscarriage, low birth weight, stillbirth and rarely congestive heart failure (58). A study by Casey *et al* found that the three-fold risk of placental abruption and a two-fold risk of preterm delivery were reported in mother with sub-clinical hypothyroidism (68). Because of THs are crucial to fetal brain and nervous system development, uncontrolled hypothyroidism especially during the first trimester can affect the baby’s growth and brain development (58, 68). 

Previous study conducted by Haddow *et al* reported that untreated hypothyroidism during pregnancy can cause a significant decreased in the intelligence quotient of children (69). Two prospective studies showed that persistent hypothyroxinemia at 12 wk of gestation was associated with an 8-10 point deficit in mental and motor function scores in infant offspring compared to children of mothers with normal thyroid function and the first trimester maternal FT4 was a significant predictor of orientation scores. But the developmental scores were not influenced by further declines in maternal FT4 at 24 and 32 wk gestation (70, 71).

**Management of hypothyroidism in pregnancy** **: **Hypothyroidism is treated with synthetic TH called thyroxine (LT4) which is identical to the natural T4. Pregnant women with pre-existing hypothyroidism will need to increase their pre-pregnancy dose of T4 to keep normal thyroid gland function. Thyroid gland function should be monitored every 6 to 8 weeks during pregnancy. Synthetic T4 is safe and necessary for the well-being of the fetus if the mother has hypothyroidism (72). Asymptomatic pregnant women should be routinely screened for hypothyroidism and patients with subclinical hypothyroidism should be treated to ensure a healthy pregnancy (73). A prospective intervention trial study found that treatment of hypothyroidism by LT_4_ reduces the risk of adverse maternal and fetal outcomes (60).

Dietary Supplements like iodine is an important mineral for a mother in pregnancy, because the thyroid uses iodine to make TH. During pregnancy, the baby gets iodine from the mother’s diet. A woman requires more iodine when they are pregnant (74). Choosing iodized salt supplemented with I^-^ over plain salt and prenatal vitamins containing I^-^ will ensure dietary supplement. A prospective study conducted by Murcia et al found that women took mean dose of 100-149 ug/day during pregnancy, and their children showed a two point decreased in PDI compared with the children of mother taking <100 ug/day. This difference was greater when mother took >150 ug/day with a decreased of 5.5 points (75).

## Conclusion

Different studies have showed that thyroid dysfunction is common in pregnancy. The major causes for this dysfunction is hormonal and metabolic changes during pregnancy leading to profound alterations of the biochemical parameters of the thyroid function. Understanding the normal physiological adaptation of the pituitary-thyroidal axis in pregnancy enables us to manage cases of thyroid dysfunction. Uncorrected thyroid function in pregnancy has adverse effects on fetal and maternal well-being. Thyroid disease usually affects females of the reproductive age group and caring for these women during pregnancy requires careful monitoring of both the mother and the fetus. Appropriate diagnosis, care and management of thyroid dysfunction in the pre-pregnancy, pregnancy and post-pregnancy periods are important to minimize the risk of complications, long-term effects of the mother and fetus.

## Recommendation

Clinical evaluation of the patient’s symptoms as well as laboratory testing should be done carefully to assess thyroid function during pregnancy. It is recommended that all pregnant mothers should be given thyroid function test and those who have thyroid dysfunction should be provided appropriate treatment and follow up until there will have normal thyroid function. Developmental follow-up of the babies of thyroid dysfunction mothers is also recommended in order to identify cognitive and other deficiencies as early as possible and provide appropriate management. 

## Conflict of interest

The authors declare that they have no competing interest.
